# The lncRNA DLX6-AS1/miR-16-5p axis regulates autophagy and apoptosis in non-small cell lung cancer: A Boolean model of cell death

**DOI:** 10.1016/j.ncrna.2023.08.003

**Published:** 2023-08-10

**Authors:** Shantanu Gupta, Daner A. Silveira, José Carlos M. Mombach, Ronaldo F. Hashimoto

**Affiliations:** a Instituto de Matemática e Estatística, Departamento de Ciência da Computação, Universidade de São Paulo, Rua Do Matão 1010, São Paulo, SP, 05508-090, Brazil; b Children's Cancer Institute, Porto Alegre, Rio Grande do Sul, Brazil; c Departamento de Física, Universidade Federal de Santa Maria, Santa Maria, RS, 97105-900, Brazil

**Keywords:** lncRNA DLX6-AS1, miR-16, BMI1, Autophagy, Apoptosis, Stress signals, NSCLC

## Abstract

Long non-coding RNA (lncRNA) distal-less homeobox 6 antisense RNA 1 (DLX6-AS1) is elevated in a variety of cancers, including non-small cell lung cancer (NSCLC) and cervical cancer. Although it was found that the microRNA-16-5p (miR-16), which is known to regulate autophagy and apoptosis, had been downregulated in similar cancers. Recent research has shown that in tumors with similar characteristics, DLX6-AS1 acts as a sponge for miR-16 expression. However, the cell death-related molecular mechanism of the DLX6-AS1/miR-16 axis has yet to be investigated. Therefore, we propose a dynamic Boolean model to investigate gene regulation in cell death processes via the DLX6-AS1/miR-16 axis. We found the finest concordance when we compared our model to many experimental investigations including gain-of-function genes in NSCLC and cervical cancer. A unique positive circuit involving BMI1/ATM/miR-16 is also something we predict. Our results suggest that this circuit is essential for regulating autophagy and apoptosis under stress signals. Thus, our Boolean network enables an evident cell-death process coupled with NSCLC and cervical cancer. Therefore, our results suggest that DLX6-AS1 targeting may boost miR-16 activity and thereby restrict tumor growth in these cancers by triggering autophagy and apoptosis.

## Highlights

• The Boolean Model describes the lncRNA DLX6-AS1/miR-16 axis in NSCLC.
• The model represents crosstalk between autophagy and apoptosis signaling pathways under stress signals.
• The model predicts a positive circuit involving ATM/miR-16/BMI1 guiding autophagy and apoptosis.
• An additional HeLa cell line was included in the model's generalization.
• This approach suits gain/loss-of-function studies for inducing specific phenotypes like autophagy or apoptosis.

## 1 Introduction

MicroRNAs (miRNAs) are well-studied master regulators of gene expression that play an important role in basic biological processes [1]. Recent findings imply that modifying the expression of miRNAs might correspond to Stress signals challenges (such as radiation, chemotherapy, reactive oxygen species (ROS), and DNA damage signaling) [1]. Equally, Long non-coding RNAs (LncRNAs) are non-coding RNAs (ncRNAs) with a total length of more than 200 nucleotides [2]. LncRNAs contribute to a wide range of cellular processes, including tumor growth and development, the epithelial-to-mesenchymal transition (EMT) process, and genome stability [2]. Additionally, by trapping miRNAs, lncRNAs can indirectly control the levels of mRNA production. They may also function as sponges [3] or decoys [4] or as competitive endogenous RNAs (ceRNAs) [5].

In this context, the study conducted by Wu et al. [6] has verified DLX6-AS1's role in promoting the progression of non-small cell lung cancer (NSCLC) through the regulation of the miR-16/BMI1 pathway. Specifically, Wu et al. observed that DLX6-AS1 was overexpressed in NSCLC, while miR-16 was downregulated in the same cell line [6]. Moreover, they demonstrated that the downregulation of DLX6-AS1 leads to the initiation of miR-16 expression. Additionally, they found that miR-16 directly targets BMI1, resulting in the inhibition of BMI1 expression and induction of apoptosis in NSCLC [6]. Interestingly, BMI1 has been linked to the activation of the DNA damage response (DDR) checkpoint through its inhibitory effect on the ATM serine/threonine kinase (ATM) pathway [7]. Specifically, overexpression of BMI1 has been shown to decrease the phosphorylation of H2A histone family member X (H2AX) and ATM in MCF7 and DU145 cells, thereby affecting DDR [7]. Conversely, the knockdown of BMI1 accelerates DDR by activating H2AX and ATM, ultimately leading to DDR checkpoint activation [7]. Similarly, another study suggests that suppression of BMI1 promotes autophagic cell death by inducing DDR and ATM in cancer cells [8]. Furthermore, inhibition of BMI1 significantly hinders the activation of the AKT serine/threonine kinase 1 (AKT) pathway, which is known to induce autophagy [8]. In line with NSCLC, emerging evidence indicates the cooperative role of ATM in triggering autophagy during DDR [9,10]. Interestingly, ATM serves as the key regulator of miR-16 activity in DDR [11].

**Fig. 1:**
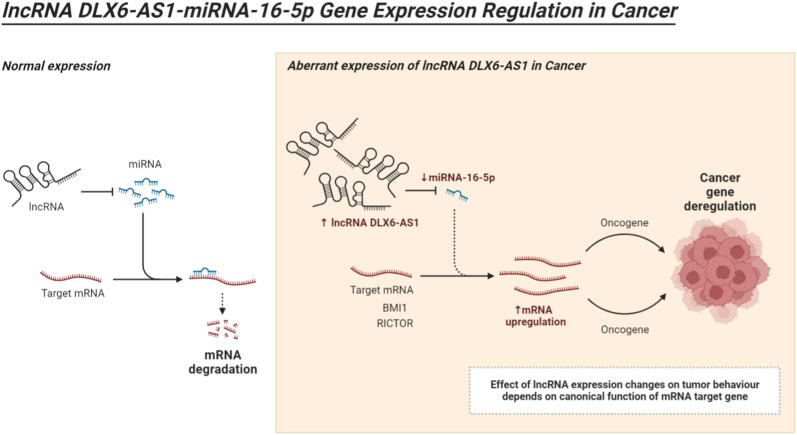
**The normal expression of lncRNA and the influence of lncRNA expression changes on tumor growth and development rely on the canonical function of the mRNA target gene.** The expression of the oncogenes RPTOR independent companion of MTOR complex 2 (RICTOR), Polycomb complex protein BMI-1 (BMI1), and others is activated as a result of abnormal DLX6AS1 expression, which deregulates the activation of miR-16 and promotes the development of cancer.

The regulatory role of miR-16 in various signaling pathways involved in stress-induced cell cycle arrest, senescence, and apoptosis is widely recognized. However, recent investigations have shed light on its potential involvement in autophagy signaling. Huang et al. [12] established the role of miR-16 in autophagy activation by targeting Mammalian target of rapamycin complex 2 (mTORC-2) within this framework. Additionally, they observed that overexpression of miR-16 in HeLa cells prevents G1 arrest and apoptosis [12]. Interestingly, according to Xie et al. [13], DLX6-AS1 was shown to be overexpressed in cervical cancer, similar to NSCLC, whereas miR-16 was found to be downregulated in the same cell line [13]. Moreover, Xie et al. [13] demonstrated that DLX6-AS1 downregulation triggers miR-16 expression. Furthermore, Xie and colleagues [13] demonstrated that cAMP regulated phosphoprotein 19 (ARPP19) is a direct target of miR-16, suggesting that increased miR-16 production suppresses ARPP19 and enhances apoptosis in cervical cancer. However, aberrant expression of DLX6-AS1 disrupts miR-16 activation, thereby promoting the activation of oncogenes such as RICTOR and BMI1, contributing to cancer progression (see Fig. 1).

The basic goal of describing a complex system, such as the DLX6-AS1/miR-16 signaling in stress signals, is to construct a model that can quantitatively predict the result of each component. Therefore, Boolean network modeling is the most effective way to merge existing data into a logical framework that is congruent with experimental results. Signaling molecules (signaling proteins and noncoding RNAs like lncRNAs and miRNAs) are often called nodes, and the relationships between them are called edges [14,15]. Cell fates correspond to model attractors (endpoints points or cyclic attractors), and their identification and accessibility attributes lend themselves well to this approach [[16], [17], [18]]. Furthermore, evaluating closed paths (parallel to feedback loops in the continuous model) connecting two or more nodes in a network could serve as regulatory circuits impacting network dynamics, which is another feature of the Boolean network [19,20]. More information on Boolean modeling can be found in the Methods Section.

**Fig. 2:**
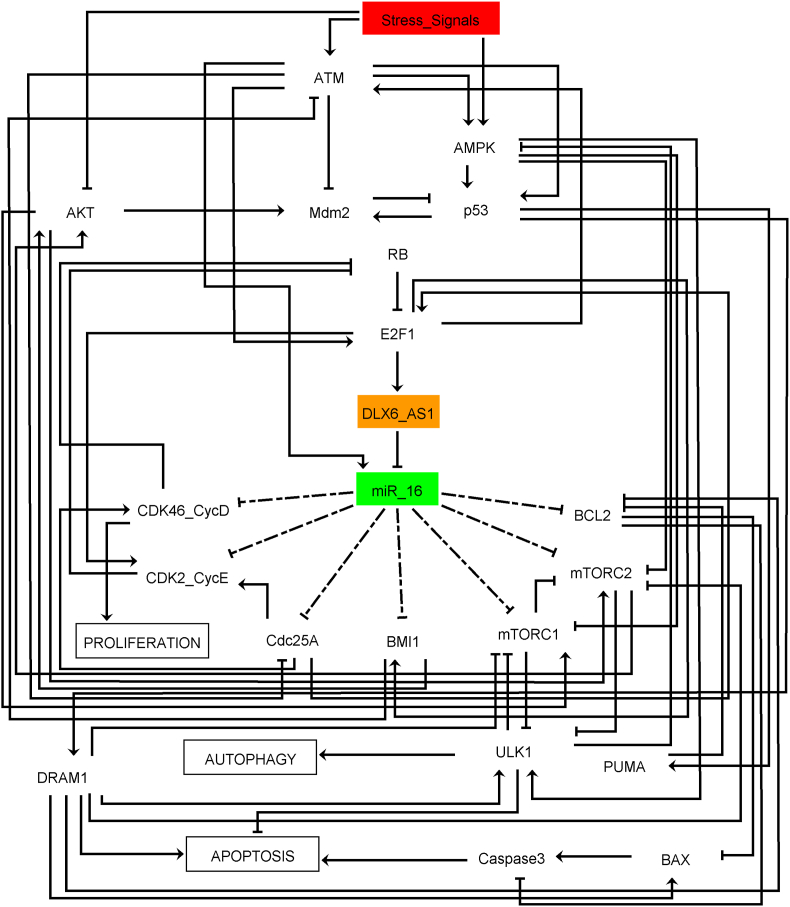
**The network of autophagy and apoptosis regulation by the DLX6-AS1/miR-16 axis in cancer cells**. Direct black edges that end in an arrowhead indicate positive interactions or regulatory relationships. Direct black edges that end in a hammerhead indicate negative interactions or regulatory relationships. While, dashed edges that end in a hammerhead represent targets of miR-16. The color of the nodes represents their function, as follows: signaling proteins are in white nodes, lncRNA DLX6-AS1 in a rectangular node (in orange), and miR-16 in a rectangular node (in green). The stress signals are represented by the input rectangle node in red. Proliferation, Autophagy, and Apoptosis are the model outputs shown in white at the rectangular nodes. The full names of network components corresponding to each node and biological justification for the edges with their regulators are provided in Supplementary Table S1.

In light of the evidence described aforementioned, we proposed a dynamic Boolean model (see Fig. 2) for controlling the processes of autophagy and apoptosis in cancer cells by the DLX6-AS1/miR-16 axis in stress signals.

## 2 Methods

### 2.1 The topology of the gene regulation network in NSCLC cells and the combination of public databases/tools

In constructing gene regulatory networks for non-coding RNAs (ncRNAs) DLX6-AS1 and miR-16, we solely relied on PubMed studies and databases such as BioGRID 3.5 (https://thebiogrid.org/) [21]. The objective was to identify genes or proteins that were targeted by these ncRNAs (Fig. 2). For example, the targets of miR-16 such as Cyclin-dependent kinases 4 and 6 complex/CyclinD1 (CDK4/6-Cyclin D), Cyclin-dependent kinase 2/CyclinE2 (CDK2/Cyclin E), Cell division cycle 25 A (Cdc25 A), BCL2 apoptosis regulator (BCL2), BMI1, Mammalian target of rapamycin complex 1 (mTORC1), and mTORC2. (see the reviewed by Ghafouri-fard [22]). On the other hand, DLX6-AS1 acts as a target for miR-16. To achieve this, we used publicly available datasets such as Target Scan Human 7.2 (http://www.targetscan.org/vert_72/) [23] to ensure accurate and comprehensive analysis.

The construction and simulation of the Boolean model were performed using GINsim 3.0.0 b [24], a Java-based tool designed for academic use (http://www.ginsim.org/downloads). GINsim algorithms detect all attractors in both the wild-type systems (unperturbed Boolean model) and mutant instances [24]. The model file can be accessed from the "Code Availability" section.

### 2.2 Dynamic boolean network model, rules, and simulations based on PubMed literature

The Boolean technique is grounded in the examination of a regulatory graph, whereby every node represents a signaling component and each straight edge (or arc) represents an activation or inhibition between two nodes. Nodes are Boolean variables that only allow "0″ and "1″ values, corresponding to "active" and "inactive" states. Each node in the network is given a logical rule based on the interpretation of the biochemical information, which governs its activation level in relation to the location of its regulators [25].

The biological interconnections stated in the gene regulatory network (Fig. 2) were encoded into Boolean rules to establish a Boolean network of ncRNAs (DLX6-AS1 and miR-16). These Boolean rules for governing nodes are based on PubMed biological literature and may be found in Supplementary Table S1. The classic Boolean operators "AND," "OR," and "NOT" were employed to build these rules. The key outcome of simulations utilizing a Boolean network is attractors. A state transition graph (STG) allows us to know the dynamical functioning of a Boolean model. Every node in this graph reflects the present state of the network variables, and the arcs describe conversions between these states. The STG accommodates all potential trajectories from such an initial state to a final state. Stable states (or fixed points) are terminal nodes with no outgoing edges, whereas a cyclic state is considered a series of transitions locked within a fixed group of states in the STG. Asynchronous updates were considered to account for state updates, which may reflect the non-deterministic behavior exhibited in molecular networks [26]. Additionally, negative and positive circuits (also known as feedback loops) govern the dynamics of a gene regulatory network. Negative circuits can stimulate oscillations, while positive circuits are in charge of multi-stable dynamics. Furthermore, this method allows for in silico gain-of-function (GoF) or loss-of-function (LoF) perturbations, in which we constrain node values to be "active" or "inactive", respectively [26,27]. This technique facilitates the investigation of the impact of individual nodes on network dynamics and the resulting phenotype [24,25].

### 2.3 Molecular mechanisms underlying stress-induced cell death events, including autophagy and apoptosis

In response to stress signals, cell death such as apoptosis or autophagy occurs at both (the G1/S and G2/M) cell cycle checkpoints [28]. In this section, we describe briefly the key direct molecular interactions reported in the literature that form our Boolean network, which includes the DLX6-AS1/miR-16 axis.

Stress signals can cause DNA double-strand breaks, which can activate the ATM and tumor protein p53 (p53) pathways [29]. ATM may directly promote miR-16 expression [11]. Conversely, E2F transcription factor 1 (E2F1) induces the expression of DLX6AS1 [30], an upstream negative regulator of miR-16 [6]. Interestingly, DNA damage causes a particular activation of E2F1 accumulation that is reliant on ATM kinase activity; in turn, E2F1 interacts with ATM to influence p53 activity [31]. Additionally, disruption of DLX6-AS1 leads to the upregulation of miR-16 [6]. Upregulated miR-16, exerts direct targeting on several pivotal proteins, encompassing CDK4/6-Cyclin D, CDK2/Cyclin E, Cdc25 A, BCL2, BMI1, mTORC1, and mTORC2, (see the reviewed by Ghafouri-fard [22]). Specifically, it targets Cdc25 A to prevent the activation of CDK4/6-Cyclin D, CDK2/Cyclin E, and E2F1 [32]. Additionally, it directly inhibits CDK4/6-Cyclin D and CDK2/Cyclin E, thereby promoting the expression of Retinoblastoma 1 protein (RB1) [32], which is necessary to block the G1/S checkpoint through the formation of a positive circuit that mediates Cdk2-CycE/E2F1/RB1 [33]. Of note, BMI1 serves as a negative regulator of ATM [34]. Intriguingly, miR-16 enhances ATM expression by specifically targeting BMI1 [6]. Moreover, miR-16 inhibits Bcl2 [35], ultimately leading to the activation of BCL2 associated X (BAX)/Caspase 3 and directing apoptosis. Furthermore, miR-16 induces autophagy by targeting both mTORC1 [36] and mTORC2 [12], thus triggering the activation of unc-51 like autophagy activating kinase 1 (ULK1). On the other hand, activated p53 causes transcription of the E3 ubiquitin-protein ligase (Mdm2), which is its repressor [37]. The p53 activates the apoptotic regulators Bcl-2-binding component 3 (BBC3, also known as PUMA), DNA damage regulated autophagy modulator 1 (DRAM1), and BAX [38]. The concept involves the activation of Caspase 3 by BAX, which PUMA and DRAM1 regulate. It is widely established that mTORC1 and mTORC2 targeting may promote the autophagy phenotype [39]. mTORCs directly inhibit the ULK1 protein complex, which is essential for autophagy activation [40,41]. Inhibiting mTOR increases ULK1 kinase activity, which leads to autophagy induction [42]. mTORC1 and ULK1 formulate an interesting double negative circuit [[43], [44]]. Furthermore, mTORC2 and AKT are formed via a positive circuit [45].Interestingly, AMPK kinase increases AMPK-P-dependent ULK1, but activated ULK1 inhibits AMPK-P, producing a negative circuit [46]. DRAM1 is needed for apoptosis via direct activation of BAX [47]. DRAM1, on the other hand, diminishes mTORCs expression, which indirectly stimulates ULK1 [48].

Based on the fundamental interactions described above, we developed our Boolean model of cell death regulation in cancer cells.

## 3 Results

### 3.1 Endpoints of the boolean network

The network has 22 signaling components, including one miRNA (miR-16) and one lncRNA (DLX6-AS1). Stress signals are also present, as a single input with two possible states of "ON" and "OFF." Proliferation, autophagy, and apoptosis are three outputs to the model. Besides that, 85 direct connections exist between these signaling components.

**Fig. 3:**
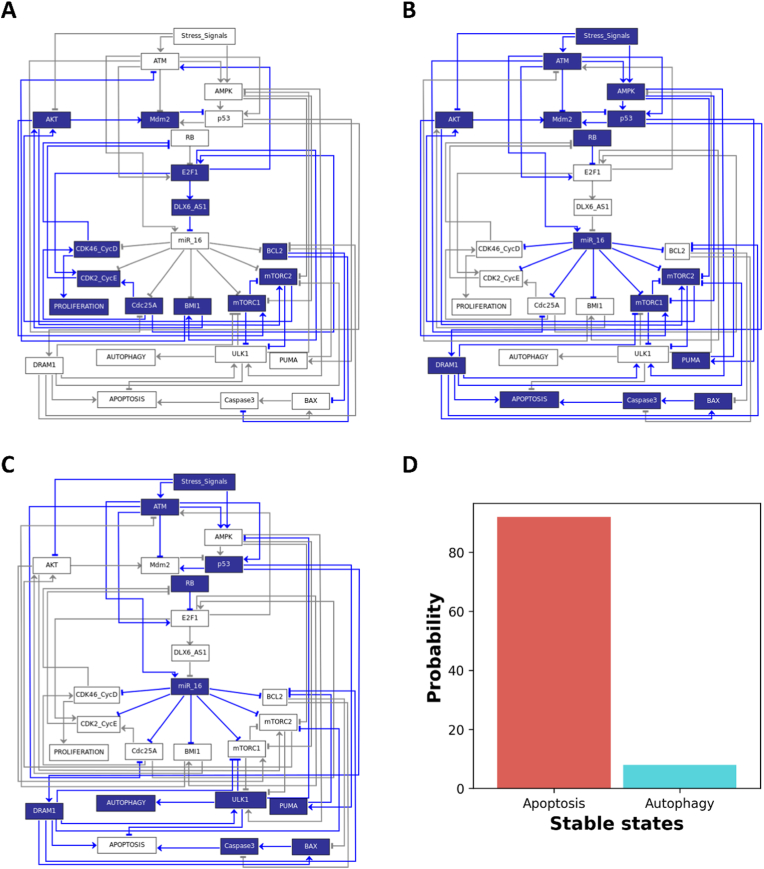
**Unperturbed dynamics of Boolean network and probabilities.** An endpoint attractor is depicted in each network illustration. The relevant molecule's ON/Activation and OFF/Inactivation states are determined by blue and white color nodes, respectively. We identified three distinct endpoints. Based on the activation of the appropriate signaling component, each endpoint represents a phenotype. In the absence of Stress-signals, we identified one endpoint. The other two endpoints arise as a result of stress signals. All of these endpoints have been defined here. (A) Proliferation phenotype is shown by the activation of cell cycle inducers in the absence of stress signals (Input). In the presence of Input has the additional two endpoints. (B) In contrast, Puma, Bax, and Caspase activity indicate an apoptotic phenotype. (C) The autophagy phenotype is defined by the activation of the ULK1 in collaboration with Puma, Bax, and Caspase. (D) Monte-Carlo simulation (100,000 Runs) was used to determine the probabilities of each endpoint or phenotype in the unperturbed dynamics of the model in response to stress signals.

We indicate the unperturbed dynamics of the network. We obtained 3 stable state points (also called endpoints). Blue and white colored nodes signify the activation and deactivation of the respective molecules, respectively. In this way, we can determine which molecules are trapped in each endpoint. There is only one input to our network called the stress signal. This input to the network can be "active" or "passive". Of the three endpoints, one endpoint, we found, occurred when the input was turned off. The remaining two endpoints we got in the presence of the input. In more detail, Fig. 3A shows a proliferative endpoint in the absence of input (when the stress signal is "non-functioning"). As can be seen, only cell cycle regulators have engaged in Fig. 3A, whereas tumor suppressors and cell cycle inhibitors are inactive. The remaining endpoints are defined by the presence of stress signals. Fig. 3B and C shows the endpoints of cell death, autophagy, and apoptosis. In more detail, Fig. 3B, illustrates the apoptotic phenotype in ULK1 deficiency, leading to the activation of DRAM1, BAX, and Caspase-3. Fig. 3C shows the autophagy phenotype triggered by ULK1 induction, as well as DRAM1, BAX, and Caspase-3. It is a non-deterministic (stochastic) network dynamics strategy in which two particular endpoints (when the input to the network is "enabled") are randomly selected from the same initial state. The probabilities of these endpoints are not necessarily equivalent. We found 11% for autophagy and 89% for apoptosis in the input using Monte Carlo simulations (100,000 runs), as shown in Fig. 3D. Interestingly, these endpoints that we found are according to the Wu et al. [6] for NSCLC, Xie et al. [13] and Huang et al. [12] for Cervical Cancer.

### 3.2 Comparison of in-silico modeling with experimental perturbations

**Fig. 4:**
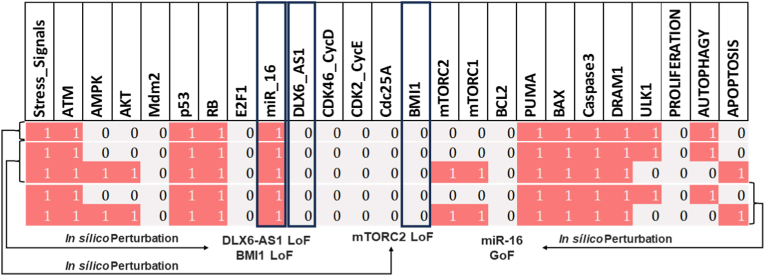
**In silico perturbations were used to validate the model through the comparison with in-vivo and in-vitro experiments reported in the literature.** The perturbations for gain-of-function (GoF) and loss-of-function (LoF) are consistent with the experiments conducted by Wu et al. [6] and Huang et al. [12]. Ectopic expression (E1) implies GoF, whereas knockdown (KO) of the same network component shows LoF. Pink cells indicate activation (ON), whereas gray cells suggest inactivation (OFF). Endpoints are identified for each of the following modeling situations: DLX6-AS1 KO, BMI1 KO, and miR-16 E1. The input Stress Signals are shown in the left column, and the model outputs are shown in the right column: proliferation, autophagy, and apoptosis. Each line indicates a single endpoint related to the input.

Additionally, we investigated whether DLX6-AS1 overexpression or miR-16 downregulation, or vice versa, influenced cell death characteristics such as autophagy and apoptosis using gain or loss of function perturbation, as suggested by Huang et al. [12] for autophagic cell death and Wu et al. [6] for apoptotic cell death. As can be seen in Fig. 4, DLX6-AS1, BMI1, and mTOR have been reported to be upregulated in NSCLC [6] and HeLa cells [12], facilitating tumor growth and migration. While miR-16 has been determined to be downregulated [6,12]. According to Wu et al. [6], DLX6-AS1 knockdown (KO) reduces proliferation and initiates apoptosis. In accordance with these findings, we discovered that knocking down DLX6-AS1 can reactivate miR-16. Interestingly, we found that DLX6-AS1 knockdown accelerates both apoptosis and autophagic cell death. We then overexpressed miR-16 and found that it suppresses proliferation by activating autophagy and apoptosis. Furthermore, Huang et al. [12] previously established that miR-16 modulates autophagy in cervical cancer by targeting mTOR2. Our findings indicate that DLX6-AS1 knockdown activates miR-16, and that activated miR-16 regulates the cell death phenotype seen in NSCLC and cervical cancer cells by Wu et al. [6], and Huang et al. [12], respectively.

### 3.3 Modulation of autophagy and apoptosis by miR-16/BMI1 axis

**Fig. 5:**
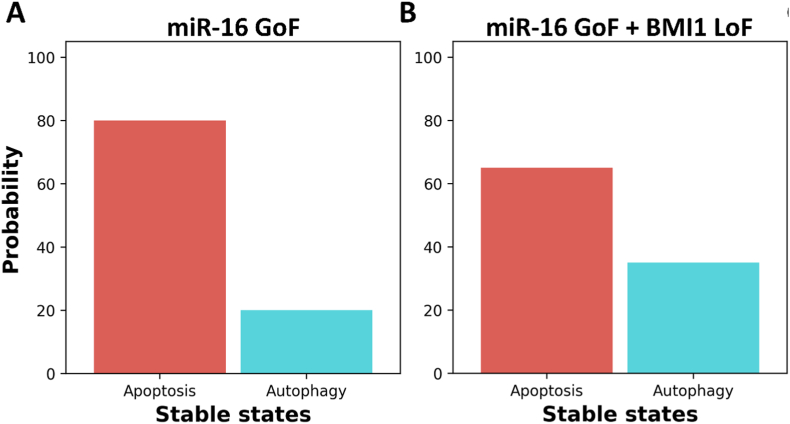
**Implications of the miR-16/BMI1 axis on cell death phenotypes.** (A). Gain of function (GoF) perturbation of miR-16 alone. (B). Gain of function (GoF) of miR-16 with loss of function (LoF) of BMI1. Each bar indicates a cell death characteristic, such as apoptosis and autophagy. For each perturbation, we have run 10.000 Monte Carlo simulations. For more detail see the "Modulation of autophagy and apoptosis by miR-16/BMI1 axis" Section.

Furthermore, we explored the implications of miR-16, and BMI1 in the modulation of autophagy and apoptosis. We intended to determine whether miR-16 overexpression could be studied either alone or in combination with BMI1 knockdown as suggested by Wu et al. [6]. This allowed us to determine which molecules had the most influence on each phenotype. We ran Monte Carlo simulations (10.000 runs) for each perturbation to achieve this. See Fig. 5, which shows our findings. MiR-16 overexpression alone induced 20% autophagy and 80% apoptosis. While, overexpression of miR-16, in concert with BMI1 knockdown, led to 35% autophagy and 65% apoptosis. As demonstrated in Fig. 5, the combination of BMI1 knockdown (KO) with miR-16 overexpression can produce higher levels of autophagic cell death than either treatment alone.

### 3.4 Molecular circuits and their dynamics

Gene regulatory networks (GRNs) are constituted of both positive and negative circuits. These circuits can capture and analyze the dynamics of biological systems. To verify that our network is able to produce some circuits. We decide to seek out more about them. GINsim only discovered eight actual biological circuits that actively influence network dynamics (see Table 1). We have one novel circuit out of these eight Table 1 highlighted in yellow), and the other seven have already received experimental recognition.

**Table 1:**
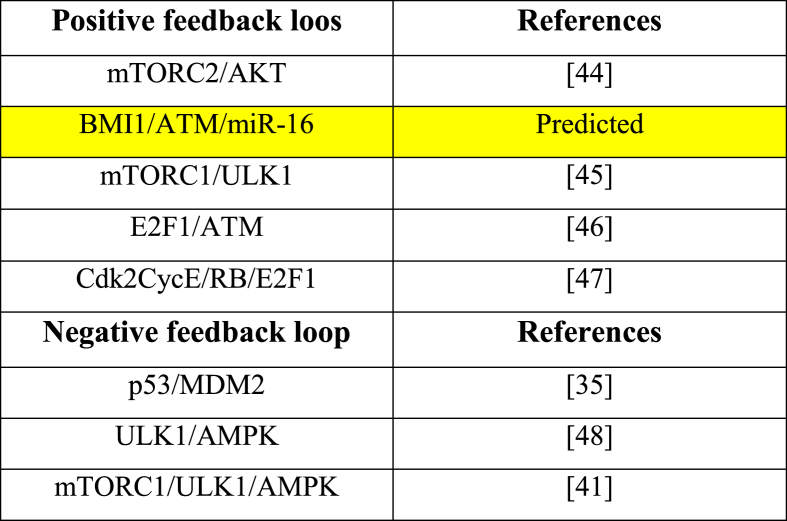
**Boolean network functional circuits and experimental obedience.** The network's predicted positive circuit is highlighted in yellow.

One of these 8 circuits is unique, BMI1/ATM/miR-16. We opted to look into this novel circuit only to establish the relevance of the DLX6-AS1/miR-16 axis in autophagy and apoptosis activation. The molecular relationships that characterize this circuit in cancer cells have been reported (Table 2). Nevertheless, the impact of this circuit on the molecular mechanisms of cell death has yet to be confirmed experimentally. Thus, we investigated to see if altering the circuit interaction may change the activation of autophagy and apoptosis. Results are summarized in Table 3. As we can see from the perturbation of the circuit between (BMI1/ATM/miR-16), whether all molecules are knocked down (KO/KO/KO) or simply BMI1 is overexpressed, it leads to proliferation. Whereas knocking it out with overexpression of ATM or miR-16 causes apoptosis. ATM knockdown causes two cases of proliferation and two cases of apoptosis. When overexpressed with miR-16, it causes three cases of apoptosis and one case of autophagy and apoptosis. MiR-16 knockdown causes two cases of proliferation and two cases of apoptosis. When overexpressed with ATM, it causes three cases of apoptosis and one case of autophagy and apoptosis.

**Table 2:** The literature is replete with evidence of biochemical interactions that characterize a biological positive circuit.

Positive Circuit	Circuit Elements	Target	Direct/Indirect Interaction	References
BMI1/ATM/miR-16	BMI1	ATM	Direct inhibition	[34]
	ATM	miR-16	Direct activation	[11]
	miR-16	BMI1	Direct inhibition	[6]

**Table 3:** **Perturbations in the newly identified positive circuit**. Ectopic expression (E1) signifies gain-of-function (GoF), whereas knockdown (KO) reflects loss-of-function (LoF).

Positive circuit	Perturbations	Phenotypes
BMI1/ATM/miR-16	KO/KO/KO	Proliferation
	E1/KO/KO	Proliferation
	KO/E1/KO	Apoptosis
	KO/KO/E1	Apoptosis
	E1/E1/KO	Apoptosis
	E1/KO/E1	Apoptosis
	KO/E1/E1	Autophagy and Apoptosis
	E1/E1/E1	Apoptosis

In addition, on this circuit, we executed edge perturbations (see Table 4). This sort of perturbation is challenging to achieve experimentally; nonetheless, it is particularly beneficial in *in-silico* analysis to evaluate circuit significance. First, we choose the interaction between the upstream node and one of the additional nodes (in this instance, miR-16 and BMI1), followed by the remaining interactions in the circuits. When the interface connecting miR-16 and BMI1 is broken, autophagy and apoptosis are abrogated. Only the circuit to which the remaining interactions belong is disrupted when they are perturbed. Any additional modification to the positive circuit above produces the same outcome, demonstrating that it governs bistability, i.e., apoptosis and autophagy.

**Table 4:** The following are the results of edge perturbations on the circuit.

Positive Circuit	Removed interactions	Abrogated phenotypes
BMI1/ATM/miR-16	BMI1/ATM	None
	ATM/miR-16	Autophagy and Apoptosis
	miR-16/BMI1	Autophagy and Apoptosis

Our results suggest the BMI1/ATM/miR-16 positive circuit might describe a novel and intriguing network of interactions between essential molecules involved in regulating autophagy and apoptosis. BMI1, a known negative regulator of ATM [34], is targeted by miR-16 [6], leading to increased miR-16 expression and subsequent inhibition of BMI1. This interaction between miR-16 and BMI1 is critical in promoting apoptosis in NSCLC and cervical cancer. Furthermore, ATM, a central player in the DNA damage response pathway, is a required controller of miR-16 activity [11], indicating a complex interplay between DNA damage signaling and cell death processes. As shown in our study, perturbing the interactions within this circuit can lead to different cellular outcomes, ranging from cell proliferation to apoptosis and even autophagy. Understanding the dynamic behavior of this circuit is vital as it provides insights into how DLX6-AS1/miR-16 axis activation may impact cell death pathways in cancer cells. Given its significance in regulating autophagy and apoptosis, targeting the BMI1/ATM/miR-16 circuit may hold therapeutic potential in treating NSCLC and cervical cancer by modulating cell survival and death responses.

## 4 Discussion

In this work, we explored the biological mechanisms underlying DLX6-AS1/miR-16 in NSCLC and cervical cancer (see Fig. 2). DLX6-AS1 is up-regulated and plays a critical role in the formation of tumors in NSCLC and cervical cancer. Conversely, miR-16 is a well-known tumor suppressor that is down-regulated in a range of malignancies, including NSCLC and cervical cancer. In fact, DLX6-AS1 regulates BMI1 through sponging miR-16 expression.

While, miR-16 overexpression suppresses proliferation in cervical cancer and NSCLC by targeting BMI1, and mTORC, and triggering apoptosis and autophagy. In more detail, Wu et al. [6] found that DLX6-AS1 was overexpressed in NSCLC whereas miR-16 was downregulated in the same cell line. Furthermore, Wu et al. [6] showed that DLX6-AS1 downregulation triggers miR-16 activation. Furthermore, Wu et colleagues [6] revealed that BMI1 is a direct target of miR-16, i.e., increased miR-16 inhibits BMI1 and promotes apoptosis in NSCLC. In this manner, Wu et al. [6] emphasized the crucial role of DLAX6-AS1 in facilitating the induction of apoptosis by the miR-16/BMI1 axis in NSCLC. Similarly, Xie et al. [13] highlighted the significance of DLAX6-AS1 in promoting miR-16-induced apoptosis in cervical cancer. Furthermore, BMI1 is linked to the AKT/mTOR pathway due to its propensity to stimulate AKT expression. It is widely accepted that mTOR inhibition initiates autophagy. Interestingly, in cervical cancer, miR-16 directly targets mTORs and controls autophagy. In this connection, Huang et al. [12] found that mTORC2 was overexpressed in cervical cancer whereas miR-16 was downregulated in the corresponding cell line. Furthermore, Huang et al. [12] found that miR-16 overexpression suppresses mTORC2 expression and causes autophagy and apoptosis. As can be seen in Fig. 4, we assessed our model, which was inspired by the works mentioned above, to determine if it could produce results equivalent to those reported in these investigations. As seen in Fig. 4, Our model exhibited good agreement with these experimental observations, supporting the validity of our approach.

In this study, we aimed to investigate the role of the DLX6-AS1/miR-16 axis in regulating BMI1 expression and its impact on autophagy and apoptosis. To achieve this, we employed gain-of-function (GoF) and loss-of-function (LoF) perturbations, followed by Monte Carlo simulations with 10,000 runs to analyze the interactions between these molecules. Specifically, we initially examined the effects of miR-16 overexpression alone and then combined it with BMI1 knockdown. The results, depicted in Fig. 5, demonstrated that the combination of miR-16 overexpression and BMI1 knockdown led to increased levels of autophagy and decreased apoptosis. These findings highlight the ability of these molecules to modulate autophagy and apoptosis in cancer cells. Overall, our study provides valuable insights into the regulatory mechanisms of the DLX6-AS1/miR-16 axis and its potential implications for modulating autophagy and apoptosis in cancer.

**Fig. 6:**
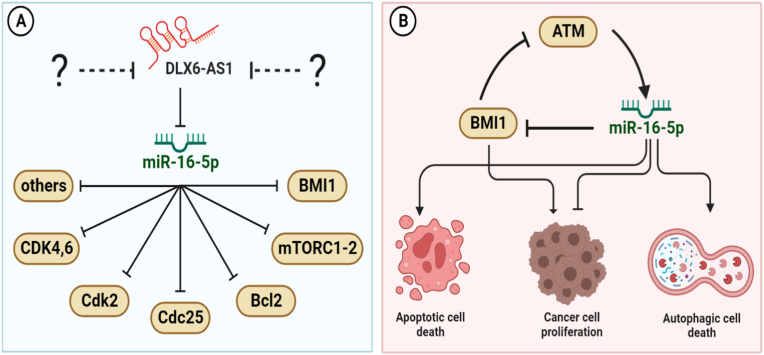
**Highlighting the key results.** A). Our findings suggest that DLX6-AS1 inhibitors (as shown in question marks) can activate miR-16 and that once triggered, miR-16 inhibits cancer progression through a cascade of events such as autophagy, apoptosis, cell cycle arrest, and senescence. The activation of the cascade can be accomplished by targeting multiple targets such as Bcl2, Cdc25, Cdk4,6, mTORC 1/2, BMI1, and others. (B). Autophagy and apoptosis activation are regulated by the double negative (positive) circuit between BMI1/ATM/miR-16. The black arrow represents activation, whereas the black hammerhead arrows represent inhibition.

According to Wu et al. [6], inhibiting DLX6-AS1 expression increases miR-16, which reduces BMI1 expression and induces apoptosis. However, it is well accepted that a single miRNA may control the gene expression of several target genes, altering biological processes via fundamentally distinct miRNA-mRNA interactions. In this scenario, DLX6-AS1 inhibitors may boost miR-16 production, and once activated, it can target its numerous target genes such as Bcl2, cdk4,6, Cdc25, mTOR 2, and so on. MiR-16 engages in a range of biological processes by targeting these proteins, including apoptosis, autophagy, senescence, cell cycle arrest, and others. For more detail see Fig. 6A. Furthermore, biological circuits play a vital role in GRNs. GRNs are, in fact, a combination of positive and negative circuits. These circuits can acquire and comprehend the dynamics within a biological system. See Table 1. In this setting, we identified a new positive (BMI1/ATM/miR-16). We also gave evidence of this circuit based on how they interact in cancer cells (for more details see Table 2). In addition, we evaluated this circuit by the perturbation of each circuit component (see Table 3). Our circuit perturbation analysis of this novel circuit reveals that the majority of scenarios favor apoptosis. Ultimately, we were capable of identifying instances that contributed to the autophagy activation (see Table 3 and Fig. 6B). Furthermore, during the perturbation investigation of this specific positive circuit, we spotted particular instances of proliferation in response to stress signals. Therefore, we suspect that these proliferative scenarios can be attributed to "drug resistance" as a consequence of stress-induced activation of DLX6-AS1, BMI1, and the G1/S checkpoint-related CDK-cyclin complex (see Table 3).

In addition, we applied edge perturbation to disrupt this circuit (see Table 4) to determine if it can alter the network dynamics. We revealed that a positive (BMI1/ATM/miR-16) circuit is capable of influencing network dynamics. For instance, in the BMI1/ATM/miR-16 positive circuit, eliminating the interaction between ATM/miR-16 abolished both autophagy and apoptosis; similarly, disrupting the interaction between miR-16/BMI1 canceled both autophagy and apoptosis.

Significantly, our findings suggest that this new circuit may be critical for controlling autophagy and apoptosis in NSCLC and cervical cancer. However, each of these noteworthy results is reliant on a discrete basis of model elements. Our method's weakness in predicting time-dependent capabilities and the precise development of expression levels over time is one of its limitations. Additionally, further research into the specific molecular processes through which the DLX6-AS1/miR-16 axis regulates cell death activation in NSCLC and cervical cancer is required. Furthermore, other lncRNAs or miRNAs may be required to govern cell death in certain malignancies. Nonetheless, it has been proven that miR-16 has implications for autophagy and apoptosis in NSCLC and cervical cancer.

**Fig. 7:**
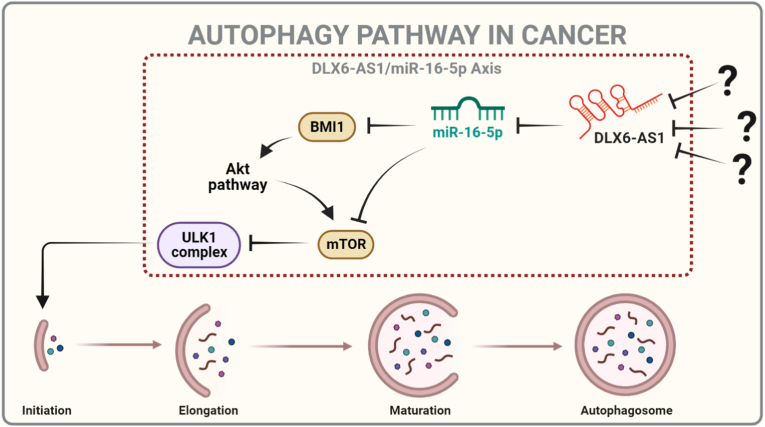
**The involvement of the DLX6-AS1/miR-16 axis in the autophagy signaling pathway.** MiR-16 can be triggered by DLX6-AS1 inhibitors (marked by question marks), and once active, miR-16 activates the autophagy signaling pathway by targeting mTOR or BMI1. As a result, the ULK1 complex becomes active. Which is required to initiate the autophagic cell death process in cancer cells. The black arrow signifies activation, whereas the black hammerhead arrows signify inhibition.

In conclusion, our model aligns with the experimental evidence regarding the regulation of autophagy and apoptosis in NSCLC and cervical cancer. We have uncovered the intricate interplay among lncRNA, miRNA, and mRNA within these processes. Furthermore, we have identified a novel positive circuit involving miR-16. The discovery of a novel role for miR-16 in controlling cell death in these malignancies is highly significant. Based on our findings, we propose that inhibiting DLX6-AS1 activity could enhance miR-16 expression, which, in turn, can induce autophagy (as shown in Fig. 7) and apoptosis in cancer cells by inhibiting BMI1 and mTORs.

## Code Availability

The model code is available in the GitHub repository (https://github.com/GuptaShan/Boolean-model-of-Cell-Death)

## Data availability

All data needed to evaluate the conclusions in the paper are present in the paper and/or the Supplementary Materials.

## CRediT authorship contribution statement

**Shantanu Gupta:** Conceptualization, Formal analysis, Investigation, Methodology, Project administration, Software, Validation, Visualization, Writing – original draft, Writing – review & editing. **Daner A. Silveira:** Conceptualization, Formal analysis, Investigation, Methodology, Software, Validation, Visualization, Writing – original draft, Writing – review & editing. **José Carlos M. Mombach:** Conceptualization, Formal analysis, Investigation, Methodology, Project administration, Resources, Software, Supervision, Validation, Visualization, Writing – original draft, Writing – review & editing. **Ronaldo F. Hashimoto:** Conceptualization, Formal analysis, Investigation, Methodology, Project administration, Resources, Software, Supervision, Validation, Visualization, Writing – original draft, Writing – review & editing.

## Declaration of competing interest

The authors declare that they have no known competing financial interests or personal relationships that could have appeared to influence the work reported in this paper.
