# Supplementary material for: The lncRNA DLX6-AS1/miR-16-5p axis regulates autophagy and apoptosis in non-small cell lung cancer: A Boolean model of cell death
Source: Noncoding RNA Res. 2023 Aug 10;8(4):605–14. doi: 10.1016/j.ncrna.2023.08.003 (PMC10520667; doi:10.1016/j.ncrna.2023.08.003)
Supplement: Multimedia component 1 [file mmc1.pdf]

Supplementary Table S1

The lncRNA DLX6-AS1/miR-16-5p Axis Regulates Autophagy and Apoptosis in Non-Small Cell Lung Cancer: A Boolean Model of Cell Death

Author Information

Shantanu Gupta[1, *], Daner A. Silveira[2], José Carlos M. Mombach[3], and Ronaldo F. Hashimoto[1,*]

Affiliations

[1] Instituto de Matemática e Estatística, Departamento de Ciência da Computação, Universidade de São Paulo, Rua do Matão 1010, 05508-090, São Paulo - SP, Brasil

[2] Children's Cancer Institute, Porto Alegre, Rio Grande do Sul, Brazil

[3] Departamento de Física, Universidade Federal de Santa Maria, Santa Maria 97105-900, RS, Brazil

Corresponding author

[*]Corresponding to: Shantanu Gupta (S.G) Email: shantanu.gupta@ime.usp.br ; Ronaldo F. Hashimoto (R.F.H) Email: ronaldo@ime.usp.br

Table S1. Logical rules that control node states of the model in Fig. 2 (highlighted in gray) in terms of the state of its regulators. The left-hand side lists the official names of the molecules (target node) (highlighted in cream color), while the right-hand side lists the references of each upcoming interaction to the target node.

Official names of the molecules	Target node	Interactions	Descriptions	References
**ATM serine/threonine kinase**	ATM	Stress Signals	Stress Signals directly activates ATM	PMID: 23939379
		E2F1	E2F1 directly activates ATM	PMID: 11459832
		BMI1	BMI1 directly inhibits ATM	PMID: 21383063
	Rule	Stress_Signals AND (E2F1 OR NOT BMI1)		ATM can be activated in the presence of Stress Signals and the absence of BMI1 or in the presence of E2F1.
**AMP activated protein kinase**	AMPK	Stress_Signals	Stress Signals directly activates AMPK	PMID: 25312480
		ATM	ATM can directly activate AMPK	PMID: 23632475
		ULK1	ULK1 inhibits AMPK	PMID: 30860465
	Rule	(ATM OR Stress_Signals) AND NOT ULK1		AMPK activated, In the presence of ATM OR Stress Signals AND In the absence of ULK1.
**AKT serine/threonine kinase 1**	AKT	Stress_Signals	Stress signals inhibits AKT	PMID: 21343617
		mTORC2	mTORC2 activates AKT	PMID: 35168143
		BMI1	BMI1 directly activates AKT	PMID: 26840020
	Rule	NOT Stress_Signals OR mTORC2 OR BMI1		Activation of AKT, In the absence of Stress Signals OR In the presence of mTORC2 AND BMI1.
**E3 ubiquitin protein ligase homolog protein**	Mdm2	p53	p53 positively interact with MDM2	PMID: 26200271
		AKT	AKT induces Mdm2 expression	PMID: 33468664
		ATM	ATM inhibits Mdm2 expression	PMID: 16601750
	Rule	(p53 AND AKT) OR NOT ATM		Mdm2 activated in the presence of p53 OR in the absence of ATM AND In the presence of AKT.

**Tumor supressor p53 protein**	p53	ATM	ATM activates p53	PMID: 16601750
		Mdm2	Mdm2 inhibits p53	PMID: 16601750
		AMPK	AMPK can activate p53	PMID: 24190973
	Rule	ATM AND (NOT Mdm2 OR AMPK)		p53 can be activated in the presence of ATM and in the absence of Mdm2 or In the presence of AMPK.
**Retinoblastoma 1 protein**	RB	CDK46_CycD	CDK46-CycD directly inhibits RB	PMID: 12362273
		CDK2_CycE	CDK2-CycE directly inhibits RB	PMID: 12362273
	Rule	NOT CDK46_CycD AND NOT CDK2_CycE		RB can be activated in the absence of CDK46-CycD and CDK2-CycE.
**microRNA-16**	miR-16	ATM	ATM can directly activate miR-16	PMID: 20668064
		DLX6-AS1	DLX6-AS1 directly inhibits miR-16 expression	PMID: 35116677
	Rule	ATM AND NOT DLX6-AS1		miR-16 can be activated in the presence of ATM AND in the absence of DLX6-AS1.
**Long non-coding RNA (lncRNA) distal-less homeobox 6 antisense RNA 1**	lncRNA DLX6 AS1	E2F1	E2F1 induces DLX6-AS1 expression	PMID: 32951317
	Rule	E2F1		DLX6-AS1 can be activated in the presence of E2F1.
**Polycomb complex protein BMI-1**	BMI1	E2F1	E2F1 activates BMI1	PMID: 16582100
		miR-16	miR-16 inibits BMI1 expression	PMID: 35116677
	Rule	E2F1 *AND NOT* miR-16		BMI1 can be activated in the presence of E2F1 AND in the absence of miR-16.
**Cell division cycle 25A**	Cdc25A	ATM	ATM directly inhibits Cdc25A	PMID: 22263797
		miR-16	miR-16 inhibits Cdc25A expression	PMID: 19536137
	Rule	*NOT* ATM *AND NOT* miR-16		Cdc25A can be activated in the absence of ATM and in the absence of miR-16.
**Cyclin-dependent kinases 4 and 6 complex/Cyclin**	CDK46_CycD	Cdc25A	Cdc25A activates CDK46-CycD	PMID: 28192398
		miR-16	CDK46-CycD is a direct target of miR-16	PMID: 18701644

**D1**	Rule	Cdc25A *AND NOT* miR_16		CDK46-CycD can be activated in the presence of Cdc25A and the absence of miR-16.
**Cyclin-dependent kinase 2/CyclinE2**	CDK2_CycE	Cdc25A	Cdc25A activates CDK2-CycE	PMID: 22263797
		E2F1	E2F1 Activates CDK2-CycE	PMID: 29754146
		miR-16	miR-16 directly inhibits CDK2-CycE	PMID: 18701644
	Rule	Cdc25A *AND* E2F1 *AND NOT* miR-16		CDK2-CycE can be activated in the presence of Cdc25A and the presence of E2F1 and the absence of miR-16.
**Serine/threonine-protein kinase mTOR**	mTORC1	AKT	AKT induces mTORC1 expression	PMID: 32023262
		ULK1	ULK1 inhibits mTORC 1 expression	PMID: 21795849
		DRAM1	DRAM1 directly inhibits mTORC1	PMID: 30902093
		miR-16	miR-16 directly inhibits mTORC1	PMID: 26538392
		AMPK	AMPK directly inhibits mTORC1	PMID: 35745143
	Rule	AKT OR NOT ((ULK1 AND DRAM1 AND miR_16) OR AMPK)		mTORC1 can be activated, In the presence of AKT AND in the absence of ULK1 AND DRAM1 AND miR-16 OR AMPK.
**Target of rapamycin complex 2 subunit MAPKAP1**	mTORC2	miR-16	miR-16 directly inhibits mTORC2	PMID: 25945419
		AKT	AKT activates mTORC2	PMID: 26235620
		DRAM1	mTORC2 is inhibited by DRAM1	PMID: 22525272
		mTORC1	mTORC1 inhibits mTORC2 activity	PMID: 29232655
		AMPK	mTORC2 is a direct target of AMPK	PMID: 19625624
	Rule	NOT miR_16 OR AKT OR NOT (DRAM1 OR mTORC1 OR AMPK)		mTORC2 can be activated, In the absence of miR-16 OR in the presence of AKT OR In the absence of DRAM1 OR mTORC1 OR AMPK.
**BCL2 apoptosis regulator**	BCL2	PUMA	PUMA directly inhibits BCL2	PMID: 30250075
		DRAM1	DRAM1 directly inhibits BCL2	PMID: 16839881
		miR-16	BCL2 is a direct target of miR-16	PMID: 16166262

	Rule	*NOT* PUMA *AND NOT* miR-145		BCL2 can be activated in the absence of PUMA and the absence of miR-16 and in the absence of DRAM1.
**BCL2 associated X, apoptosis regulator**	BAX	BCL2	BCL2 directly inhibits BAX	PMID: 23173842
		DRAM1		PMID: 16839881
	Rule	*NOT* BCL2 AND DRAM1		BAX can be activated in the absence of BCL2 and in the presence of DRAM1.
**E2F transcription factor 1**	E2F1	RB	RB directly inhibits E2F1	PMID: 23967231
		Cdc25A	Cdc25A activates E2F1 expression	PMID: 22263797
		ATM	ATM activates E2F1	PMID: 11459832
	Rule	NOT RB OR (Cdc25A AND ATM)		E2F1 can be activated in the absence of RB and the presence of Cdc25A and the presence of ATM.
**Caspase-3**	Caspase3	BCL2	BCL2 inhibits Caspase3	PMID: 16297711
		BAX	BAX enhances Caspase3 activity	PMID: 26395559
	Rule	*NOT* BCL2 *AND* BAX		Caspase3 can be activated in the absence of BCL2 and the presence of BAX.
**BCL2 binding component 3**	PUMA	p53	p53 induces PUMA expression	PMID: 14676844
	Rule	p53		PUMA can be activated in the presence of p53.
**DNA damage-regulated autophagy modulator protein 1**	DRAM1	p53	p53 induces DRAM1 expression	PMID: 16839881
	Rule	p53		DRAM1 can be activated in the presence of p53.
**Serine/threonine-protein kinase ULK1**	ULK1	DRAM1	DRAM1 can activates ULK1	PMID: 30902093
		AMPK	AMPK can activates ULK1	PMID: 22025673
		mTORC2	mTORC2 inhibits ULK1	PMID: 20083114
		mTORC1	mTORC1 inhibits ULK1	PMID: 23524951
	Rule	(DRAM1 OR AMPK) AND NOT mTORC2 AND NOT mTORC1		ULK1 can be activated, In the presence of DRAM1 OR In the presence of AMPK

				AND in the absence of mTORC2 AND in the absence of mTORC1.
**PROLIFERATION**	Proliferation	CDK46_CycD	CDK46-CyclinD induces cancer proliferation	PMID: 34395246
	Rule	CDK46_CycD		PROLIFERATION can be activated in the presence of CDK 46-CyclinD.
**AUTOPHAGY**	Autophagy	ULK1	ULK1 is a marker of AUTOPHAGY Activation	PMID: 23685627
	Rule	ULK1		AUTOPHAGY can be activated in the presence of of ULK1.
**APOPTOSIS**	Apoptosis	Caspase3	Caspase 3 can induce Apoptosis	PMID: 10200555
		DRAM1	DRAM1 can induces Apoptosis	PMID: 25633293
		ULK1	ULK1 inhibits Apoptosis	PMID: 30166400
	Rule	(Caspase3 OR DRAM1) AND NOT ULK1		APOPTOSIS can be activated in the presence of Caspase3 OR DRAM1 AND in the absence of ULK1.
